# Prevalence of Diabetes Among First-Time Ophthalmology Patients at a Nonprofit Hospital in Mexico

**DOI:** 10.3390/diagnostics15222922

**Published:** 2025-11-19

**Authors:** Valeria Sánchez-Huerta, Mary Lady González Suriel, Héctor Randolph, María José Barragán Álvarez, Benjamin Aleman-Castilla

**Affiliations:** 1 Asociación para Evitar la Ceguera en México I.A.P. (APEC), Calle Vicente Garcia Torres 46, San Lucas, Coyoacan, Mexico City 04030, Mexico; 2 Economic Environment Department, IPADE Business School (IPADE), Calle Floresta 20, Claveria, Azcapotzalco, Mexico City 02080, Mexico; 3 ALTTRAC, Atizapan de Zaragoza, Mexico City 52938, Mexico

**Keywords:** diabetes, benefit–cost analysis, SROI, nonprofit organizations, Mexico, I12, I31, I38, L31, D61

## Abstract

**Background/Objectives**: Diabetes is Mexico’s second-leading cause of death, primary cause of disability, and diabetic retinopathy (DR) associated with this disease is the leading cause of vision loss among the working population. Limited healthcare funding and inequitable access hinder diagnosis and treatment, leaving 32% undiagnosed and at risk of developing serious complications such as DR. With screening rates declining, nonprofits like the Association to Prevent Blindness in Mexico (APEC) play a crucial role in detecting diabetes and DR, reducing healthcare costs, and improving patient outcomes. **Methods**: This study analyzes data from over 25,000 first-time patients screened at APEC in 2023, providing a unique empirical resource on diabetes and DR in Mexico. Using the Social Return on Investment (SROI) approach, it evaluates program costs (medical resources, equipment, and personnel) against patient benefits. These benefits are quantified as the probability that newly diagnosed or uncontrolled diabetes patients begin treatment, thus preventing DR, weighted by the Value of Statistical Life (VSL). **Results**: Of the total screened patients, 17.2% had diabetes. Among them, 20.0% were unaware of their condition, while the remaining 80.0% knew their diagnosis. Notably, 25.8% of those who were aware of their diagnosis did not have diabetes under control. Considering all costs associated with the first-time ophthalmology patients screening program and assuming only a portion of patients would seek treatment, every peso invested by APEC has the potential to generate the equivalent to 542 pesos in patient well-being. When factoring in the subsequent costs of diabetes control treatment borne by the patients, the potential Benefit–Cost Ratio is estimated at 9:1. These results proved consistent to sensitivity analysis for key assumptions affecting the estimated benefits and costs. **Conclusions**: The study demonstrates that integrating routine diabetes screening into specialized ophthalmologic care can generate substantial social value through timely intervention, as early detection promotes better diabetes management and helps prevent complications beyond diabetic retinopathy.

## 1. Introduction

Even though diabetes is a largely preventable lifestyle disease, it remains a major public health challenge both globally and in Mexico. Diabetes is a chronic metabolic disorder that occurs when the body cannot produce enough insulin (type 1) or cannot effectively use the insulin it does produce (type 2), leading to high levels of glucose (hyperglycemia). If left untreated or poorly managed, diabetes can lead to serious complications such as cardiovascular and cerebrovascular diseases, kidney failure, blindness, and lower limb amputations [1,2,3]. Its rising prevalence and associated health risks contribute to high rates of morbidity and mortality, resulting in a substantial burden on health systems and society through diminished quality of life for individuals and escalating economic costs [4,5].

According to the 11th edition of the International Diabetes Federation Diabetes Atlas [3], an estimated 589 million adults (aged 20–79), equivalent to 11.1% of the global population in this age group, were living with diabetes in 2024. Alarmingly, diabetes and its complications were responsible for approximately 3.4 million deaths worldwide in the same year, accounting for 9.3% of global mortality in this demographic [3].

Mexico ranks eighth among the ten countries with the highest number of adults living with diabetes in 2024, highlighting the urgency of addressing this epidemic. Diabetes is currently the second leading cause of death and the primary cause of disability in the country. In 2023, it accounted for 110,059 deaths, representing 13.8% of all registered deaths nationwide [6]. Additionally, according to the National Health and Nutrition Survey 2023 [7], the burden of the disease continues to rise, as data indicate that the overall prevalence of diabetes in Mexico reached 18.4% that year, marking a 27.8% increase since 2006 [4,8].

Extensive evidence shows that timely detection and appropriate treatment can slow disease progression, prevent severe complications, and significantly improve long-term health outcomes and quality of life [5,9]. However, a persistent shortcoming in Mexico’s response to metabolic diseases is the high rate of underdiagnosis [10]. Globally, more than four in ten adults (42.8%; 251.7 million) with diabetes remain undiagnosed [3], and Mexico ranks seventh among the countries with the largest number of undiagnosed adults. The National Health and Nutrition Survey2023 data show that 32.6% of individuals living with type 2 diabetes were unaware of their condition, revealing a critical gap in early detection and timely access to treatment [8].

To address this problem, international guidelines from organizations such as the World Health Organization and the American Diabetes Association [2,11] recommend routine diabetes screening, particularly among high-risk populations. In line with these recommendations, Mexico’s Official Standard for the Prevention, Treatment, and Control of Diabetes Mellitus (NOM-015-SSA2-2010) advises screening every three years for all individuals aged 20 and older. To support this, the Ministry of Health has developed a risk scale to help identify individuals eligible for blood glucose screening [12]. However, despite the high prevalence of risk factors such as obesity and abdominal obesity in the population, screening coverage remains low. According to the National Health and Nutrition Survey data, the proportion of adults who reported having undergone diabetes screening dropped from 23.7% in 2012 to just 9.6% in 2021, revealing a concerning gap between public health policy and implementation [10].

One of the most serious and feared complications of diabetes is diabetic retinopathy (DR), a condition that occurs when chronic high blood glucose levels damage capillaries in the retina. Globally, DR affects approximately 23% of adults living with diabetes, making it a highly prevalent and burdensome complication [3]. As it advances, DR can lead to cloudy or blurry vision, faded colors, and patchy vision loss. Without timely diagnosis and treatment, DR can lead to irreversible blindness. Notably, DR is the leading cause of vision loss among the working-age population, underlining its social and economic significance. Beyond its physical consequences, vision loss severely impacts an individual’s independence, productivity, and psychological well-being [13,14,15].

In this context, the role of specialized institutions such as the Association to Prevent Blindness in Mexico (APEC) becomes vital. APEC is a nonprofit private assistance institution founded on 13 August 1918, with the mission of providing high-quality ophthalmologic care to individuals with limited economic resources. Regardless of their initial reason for seeking care, APEC performs routine screening for all first-time patients, including capillary blood glucose testing. Many patients arrive with vision problems caused by undiagnosed diabetes; thus, this proactive protocol enables both the early detection of diabetic retinopathy and the diagnosis of diabetes itself. APEC thereby plays a vital role in complementing Mexico’s public health system, not only by treating diabetic retinopathy but also contributing by identifying the underlying condition. This dual function of APEC, delivering specialized treatment and conducting systematic screening, constitutes an essential, cost-effective strategy for addressing the diabetes epidemic in Mexico. By diagnosing diabetes earlier and preventing the progression to vision-threatening complications, the hospital helps reduce long-term healthcare costs and improve patient outcomes.

The present study aims to evaluate the social and economic impact of APEC’s screening program for first-time patients, with a particular focus on its role in the early diagnosis and treatment of diabetes. Using a Social Return on Investment (SROI) framework, the analysis underscores the broader value of incorporating systematic diagnostic practices within specialized healthcare institutions. While there is extensive national and international literature on the diabetes epidemic in Mexico, there remains a significant empirical gap regarding the impact of screening programs on patients’ quality of life. By addressing this gap, the study provides new evidence to support the importance of engaging all healthcare providers, regardless of their specialty, in combating the diabetes crisis. The findings also highlight an innovative approach to early detection, positioning vision care as a gateway to identifying one of Mexico’s most pressing chronic diseases.

## 2. Materials and Methods

### 2.1. Theory of Change

**Figure 1:**
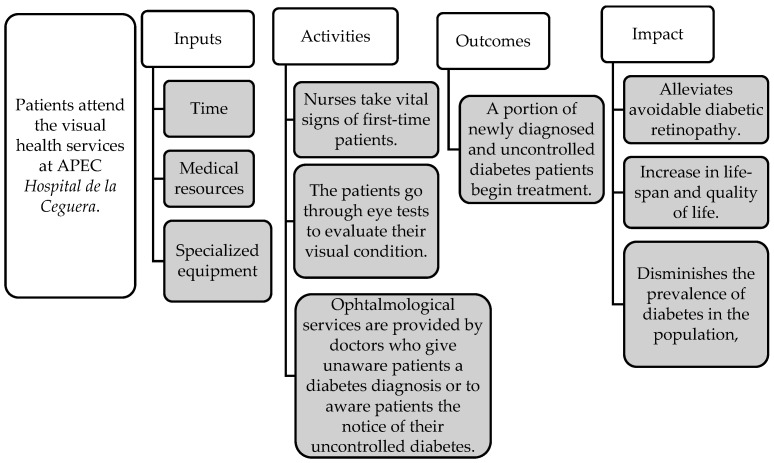
Theory of change for the APEC Hospital first-time patient screening program.

Figure 1 presents the theory of change for APEC’s screening program for all first-time patients, illustrating how key inputs translate into outputs, immediate outcomes, and ultimately, broader impacts in the lives of beneficiaries [16]. Individuals arrive at APEC for the first time seeking visual health services. As part of the hospital’s standardized intake protocol, all first-time patients undergo a comprehensive screening process in which nurses record vital signs, including casual capillary blood glucose levels. Following this, ophthalmologists conduct assessments of visual acuity, refraction, and perform pupil dilation exams. If the glucose screening indicates that a patient meets diagnostic criteria for diabetes, ophthalmologists either inform previously undiagnosed individuals of their condition or alert diagnosed patients that their diabetes is currently uncontrolled. Finally, ophthalmological treatments and services are provided.

### 2.2. Data and Statistical Analysis

This study draws on data from over 25,000 first-time patients screened at APEC Hospital in 2023, providing a unique empirical resource on the prevalence of diabetes and DR in Mexico. Data processing and statistical analyses were performed using Stata version 19.5 (StataCorp LLC, College Station, TX, USA) and Microsoft Excel 2024 (Microsoft Corporation, Redmond, WA, USA). The research was conducted in accordance with the Declaration of Helsinki, ensuring compliance with ethical principles for medical research involving human subjects. Ethical approval was obtained from the Ethics Committee of APEC Hospital (protocol code OFT-25-02, approved 6 May 2025).

As shown in Table 1, 63% of the sample were women and 37% were men. The proportion of female participants is higher than that of the national population, where women represent 51.2% according to the 2020 Institute of Statistics and Geography Census [17]. The mean age of participants was 55 years. According to the census data, 4.5% of the Mexican population falls within the 55–59-year-old age group.

Regarding factors associated with the risk of developing diabetes, the mean body weight in the sample was 70.56 kg, and the average body mass index was 28.2 kg/m^2^. This value indicates that, on average, participants fall within the overweight range, as defined by the World Health Organization, which classifies a body mass index greater than 25 as overweight [18]. The dataset includes more than 15,000 capillary blood glucose measurements, with a mean glucose level of 130 mg/dL. Notably, 12% of individuals with available glucose data exhibited levels above 200 mg/dL, suggesting possible undiagnosed or poorly controlled diabetes within this subgroup.

Patients underwent routine casual capillary blood glucose testing, measured at any time of day regardless of the time interval since the last food intake, along with a brief self-reported medical history. The analysis includes over 15,000 patients with glycemic records; however, all observations were included, as many individuals reported a previous diabetes diagnosis even without a recorded glucose measurement, and since APEC’s screening program invested resources to all participants.

To classify patients by diabetes status, four categorical variables were constructed following the guidelines established in Mexico’s Official Standard for the Prevention, Treatment, and Control of Diabetes Mellitus NOM-015-SSA2-2010 [12]:

- Uncontrolled diagnosed diabetes: individuals who reported a prior medical diagnosis of diabetes and presented a casual capillary blood glucose level ≥ 200 mg/dL.
- Controlled diagnosed diabetes: individuals with a prior medical diagnosis and a blood glucose level ≤ 200 mg/dL.
- Undiagnosed diabetes: individuals who had never received a diabetes diagnosis but presented a blood glucose level ≥ 200 mg/dL.

Total: the sum of diagnosed cases (controlled and uncontrolled) and undiagnosed cases.

**Figure 2:**
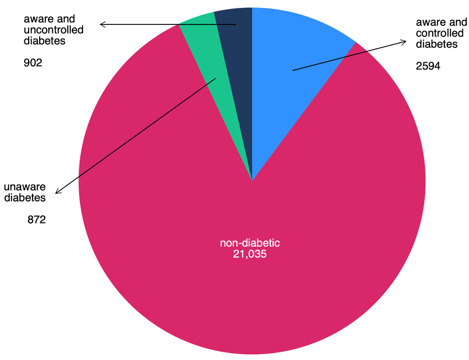
Diabetes status in first-time ophthalmology patients at APEC.

**Figure 3:**
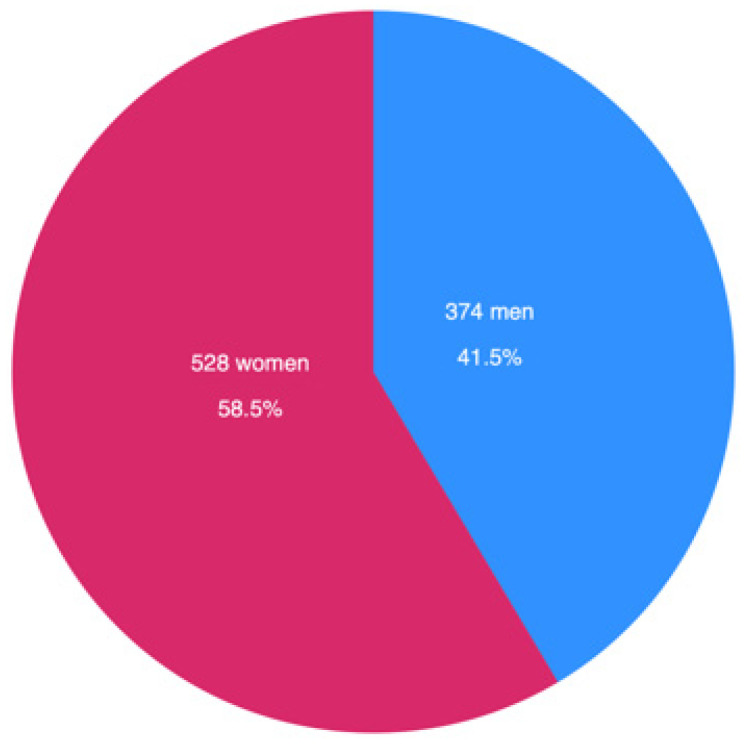
Gender distribution of uncontrolled diabetes patients.

**Figure 4:**
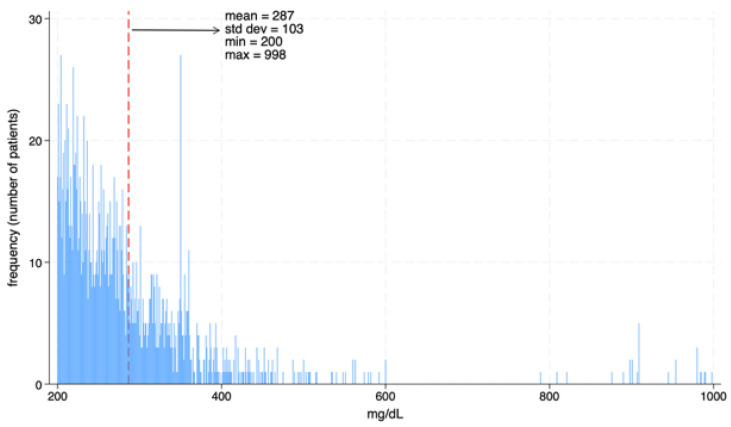
Blood glucose levels in unaware or uncontrolled diabetic patients.

**Figure 5:**
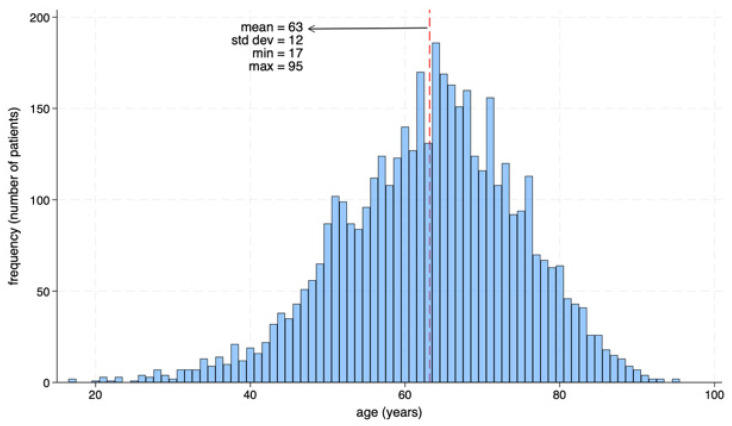
Age distribution of diabetic patients.

As illustrated in Figure 2, 17.2% of all screened patients (*n* = 4368) met the criteria for diabetes. Among them, 20.0% (*n* = 872) were unaware of their condition, while the remaining 80.0% (*n* = 3496) knew their diagnosis. Among the diagnosed population, 25.8% (*n* = 902) had uncontrolled blood glucose levels. Notably, women represented 58.7% (*n* = 512) of the undiagnosed group and 58.5% (*n* = 528) of those with uncontrolled diabetes as observed in Figure 3. In addition, undiagnosed and uncontrolled diabetic patients exhibited hyperglycemia, with a mean glucose level of 287 mg/dL in this group (Figure 4). As shown in Figure 5, the average age among patients with diabetes was 63 years, seven years higher than the mean age of the overall participant population. According to national census data, 3.8% of the Mexican population falls within the 60–64-year age group [17].

### 2.3. Inputs

Our analysis considers two main categories of costs: those borne by the institution, APEC, and those potentially incurred by patients following diagnosis. This dual perspective captures both the supply side investment in early screening and the downstream costs associated with diabetes management from the patient’s standpoint. From APEC’s perspective, the analysis includes the full range of costs associated with the standardized initial care pathway that all new patients undergo prior to diagnosis and referral to a specialty area for follow-up treatment. This pathway consists of three key stages: (1) registration, (2) vital signs screening, including capillary blood glucose testing, and (3) ophthalmologic examinations to assess visual condition, including visual acuity, refraction, and pupil dilation. Costs include supplies, medical materials, depreciation of specialized equipment, and the allocation of human resources, including both clinical and administrative staff time. Table 2 presents the summary statistics of these inputs.

**Table 2:** Summary statistics of input values for the APEC screening program per patient ^1^.

	Mean	Std. Dev
Time ^2^	$8.51	$13.16
Special equipment ^3^	$0.01	$0.02
Medical resources ^4^	$5.96	$16.66

Source: Authors’ own elaboration based on APEC administrative data. Notes: ^1^ All values are expressed in Mexican pesos. ^2^ Estimated value of hospital staff time dedicated to each patient’s first examination. ^3^ Cost of ophthalmologic and specialized equipment used during each patient’s initial examination, calculated assuming a 10% annual depreciation rate. ^4^ Cost of consumables, diagnostic supplies, and medications used throughout each patient’s evaluation.

As mentioned, in addition to the costs incurred by APEC, the analysis accounts for the potential follow-up costs from the patient’s perspective. In the baseline scenario, it is assumed that a proportion of individuals who, as a result of APEC’s screening program, are newly diagnosed with diabetes or become aware that their condition is uncontrolled will seek treatment to manage their condition, thereby generating future health expenditures. To quantify this, we estimate the present value of the diabetes-related health expenditures per person for 2021 reported by the International Diabetes Federation [19], following the annuity formulation as in Ross et al. [20] (pp. 103–106). Thus, for each patient *i*, the total costs are calculated as follows:

(1) $${Cost}_{i}={Treatment}_{i}^{0}[\frac{1}{r-g}-\;\frac{1}{r-g}\;\times {\left(\frac{1+g}{1+r}\right)}^{T}]$$

Here, ${Treatment}_{i}^{0}$ represents the diabetes-related health expenditures per person reported by the International Diabetes Federation [19], g is the negative drop-off rate, assumed to be equal to the mortality rate per 1000 inhabitants obtained from World Development Indicators [21], T denotes the number of remaining life years based on life expectancy by age group [22], and r is the social discount rate, which in our baseline scenario is set at 10% as established by the Mexican Ministry of Finance and Public Credit for evaluating investment projects [23]. All data are for the year 2021.

Finally, the expected cost per patient is weighted by the probability that they will seek treatment after being diagnosed or informed of uncontrolled diabetes. Based on observed patterns in the dataset, we assume that 74.2% of these individuals will initiate treatment. This probability was estimated as the ratio of patients with controlled diagnosed diabetes to the total number of patients who reported a prior diagnosis (both controlled and uncontrolled).

### 2.4. Outcomes

To estimate the value of outcomes from APEC’s screening program on the well-being of patients with undiagnosed or uncontrolled diabetes, we use the average disability weight (*ADW*) associated with various diabetes-related complications (*ADW* = 0.25), as reported in the Global Burden of Disease Study 2021 [24], led by the Institute for Health Metrics and Evaluation. Disability weights are numerical values that quantify the severity of health loss from specific health conditions on a scale from 0 (perfect health) to 1 (a health state equivalent to death).

To assign an economic value to the potential health improvements for these patients, the average disability weight is multiplied by the Value per Statistical Life (VSL) for Mexico. The VSL reflects people’s willingness to trade or sacrifice other resources for reductions in mortality risk. It is calculated as the marginal rate of substitution between money and mortality risk within a specific timeframe. We adopt the VSL estimate of 2.0 million U.S. dollars reported by Becerra-Pérez et al. [25], which was derived in accordance with OECD’s guidelines outlined in the Mortality Risk Valuation in Environment, Health, and Transport Policies manual [26]. This estimate was based on GDP data from Mexico’s National Institute of Statistics and Geography for 2021, a year in which the country’s economic growth was significantly impacted by the COVID pandemic. Therefore, it is important to note that the VSL may be underestimated. The VSL was then converted to Mexican pesos using the average exchange rate for 2021 (20.3 Mexican pesos per U.S. dollar) [27].

From this VSL figure, we derive the Value per Statistical Life Year (*VSLY*), which captures the annual health gains from timely diagnosis and treatment. The present value of these outcomes is calculated using a similar decreasing annuity formulation described above:

(2) $${Outcomes}_{i}={\left(ADW\times VSLY\right)}_{i}^{0}[\frac{1}{r-g}-\;\frac{1}{r-g}\;\times {\left(\frac{1+g}{1+r}\right)}^{T}]$$

Finally, these outcomes are weighted by the estimated 74.2% probability of initiating treatment after diagnosis or notification of an uncontrolled condition.

## 3. Results

### 3.1. Benefit–Cost Ratio

The benefits and costs incurred by APEC and the patients, as described earlier, were used to calculate the Benefit–Cost Ratio (BCR). This metric provides a comprehensive assessment of the program’s value for each patient i:

(3) $${BCR}_{APEC}=\;\frac{\sum_{i}{Outcomes}_{i}}{\sum_{i}{Screening\;costs}_{i}}$$

When considering only the costs associated with the first-time ophthalmology patient screening program (${BCR}_{APEC}$ in Equation (3)), and assuming that a fraction (74.2%) of newly diagnosed or uncontrolled diabetes patients would seek treatment, we obtain that every peso invested by APEC has the potential to generate the equivalent to 542 (95% CI: 518–567) pesos in patient well-being.

Furthermore, when comparing these potential benefits with the costs of the screening program, and factoring in the subsequent costs of diabetes control treatment borne by the patients who decide to do so:

(4) $${BCR}_{FULL}=\;\frac{\sum_{i}\left({Outcomes}_{i}-{Treatment}_{i}\right)}{\sum_{i}{Screening\;costs}_{i}}$$

The potential benefit–cost ratio (${BCR}_{FULL}$ in Equation (4)) for this subset of beneficiaries is estimated at 9:1 (95% CI: 8.9–9.0), meaning that for every peso invested by APEC in the screening program, 9 pesos in well-being could potentially be generated for patients that are diagnosed with diabetes and choose to pursue treatment to manage their condition.

### 3.2. Sensitivity Analysis

To evaluate the robustness of our estimates and assess how variations in key assumptions, such as treatment uptake rates, disability weights, and economic valuation metrics, might influence the estimated benefits and costs, we conduct a series of sensitivity analyses. This approach captures a broader range of potential scenarios and strengthens the reliability of our results, enabling a better understanding of the possible variability in the intervention’s outcomes and making more informed, resilient, and evidence-based policy decisions.

- Probability of initiating treatment after diagnosis or notification of uncontrolled diabetes: In the baseline scenario, it is assumed that 74.2% of patients will seek treatment after receiving a new diagnosis or being informed that their diabetes is uncontrolled, based on observed patterns in the dataset. To assess the sensitivity of our results to this parameter, we adjust this probability to 10%, 25%, 50% and 100%, as shown in case (a) of Table 3. The benefit–cost ratio ${BCR}_{APEC}$ changes by −86.5%, −66.1%, −32.6%, and +34.8%, respectively, relative to the baseline. In contrast, ${BCR}_{FULL}$ remains virtually unchanged across all these values.
- Disability weights: As previously mentioned, we used an average disability weight (*ADW* = 0.25) associated with various diabetes-related complications, based on estimates from the Global Burden of Disease Study 2021. To test the sensitivity of the BCRs to this parameter, we replace the baseline *ADW* with a lower bound of 0.17 and an upper bound of 0.32. As shown in case (b) of Table 3, this results in changes in ${BCR}_{APEC}$ of −30.4% and +30.6%, respectively. ${BCR}_{FULL}$ exhibits a similar response to these variations.
- Discount rate: We also evaluate the effect of changing the discount rate. The baseline scenario assumes a 10% discount rate, following the official guidelines for socioeconomic evaluation of investment projects issued by Mexico’s Ministry of Finance. To assess its importance, we adjust the rate to 3%, 5%, and 15%, as shown in case (c) of Table 3. These changes yield variations in ${BCR}_{APEC}$ of +80.8%, +48.6%, and −26.1%, respectively. ${BCR}_{FULL}$ remains basically constant, as the discount rate affects both the value of outcomes and the cost of treatment (Equations (1) and (2) above), while the costs of screening per person are considerably low.
- Value per Statistical Life (VSL): Finally, as explained in the Outcomes section above, our baseline scenario uses the VSL of 2.0 million U.S. dollars for Mexico, based on the estimate by Becerra-Pérez et al. [25]. In case (d) of the sensitivity analysis, we vary this parameter to 1.0 million, 1.5 million, and 2.5 million U.S. dollars. The ${BCR}_{APEC}$ responds with changes of −50.0%, −25.0%, and +25.0%, respectively, while ${BCR}_{FULL}$ follows a similar proportional pattern. This is so because the VSL enters the BCR linearly in the numerator of the calculation of total benefits.

## 4. Discussion and Conclusions

This study provides robust evidence of the social and health impact generated by integrating diabetes screening into ophthalmologic services at nonprofit institutions such as the APEC. Beyond preserving vision, APEC’s screening program can trigger a far-reaching behavioral and health effect by motivating patients to manage their diabetes, thus preventing complications that extend beyond DR. Early identification of previously undiagnosed or uncontrolled diabetes may act as a catalyst for treatment initiation and improved adherence, ultimately reducing the long-term burden of chronic complications. Based on the patterns observed in our dataset, we estimated that 74.2% of patients would seek medical care following a new diagnosis or after being informed of poor glycemic control. This assumption aligns with the findings of Basto-Abreu et al. [8], who reported that among individuals with type 2 diabetes in Mexico, 72.8% had at least one medical visit in the previous year, 92.8% were under pharmacological treatment, and 77.9% adhered to treatment regularly, with 25.8% achieving glycemic control. These results suggest that the response rate among APEC’s screened population is plausible and consistent with national care patterns, reinforcing the potential of screening programs to enhance patient engagement and disease control.

Evidence from longitudinal studies supports the notion that early detection substantially mitigates the progression and severity of DR. Olafsdottir et al. [15] demonstrated that patients whose diabetes was detected through screening had a significantly lower prevalence and severity of retinopathy than those diagnosed through conventional clinical care, even after controlling for risk factors such as disease duration and hyperglycemia. Ten years after diagnosis, the prevalence of retinopathy among the screening-detected group was 22%, compared with 51% among those clinically diagnosed. These findings highlight that screening-based detection effectively shortens the period of undiagnosed hyperglycemia, allowing for earlier preventive interventions and better long-term ocular outcomes. Consistent results have been reported in Mexico. Prado-Serrano et al. [28] found that among diabetic patients examined through clinical and angiographic retinal studies, 71% had some degree of DR, with 37% showing non-proliferative and 63% proliferative forms. The prevalence and severity of DR were markedly higher among patients with more than 15 years since diagnosis compared with newly diagnosed individuals. This temporal relationship reinforces the preventive potential of early screening, as timely management can reduce exposure to chronic hyperglycemia and delay or prevent progression to vision-threatening stages. Presumably, early detection of diabetes reduces time spent with hyperglycemia, and together with early and regular screening for retinopathy, allows more effective preventive measures against retinopathy.

The economic burden associated with DR management is substantial, encompassing both direct costs (medical care, treatments, surgeries) and indirect costs (productivity loss, disability, and informal care). Prior studies have consistently concluded that these costs escalate sharply with disease severity, posing challenges to both public and private health systems [13]. Against this backdrop, the results of our SROI analysis underscore the remarkable social value of early detection. In our baseline scenario, which includes the costs borne by both APEC and patients, the screening program achieves a potential Benefit–Cost Ratio ${BCR}_{FULL}$ of 9:1, meaning that every peso invested can generate approximately nine pesos in social value. This return remains robust across sensitivity analyses that account for variations in treatment uptake, disability weights, discount rates, and the value of a statistical life. Such a strong ratio demonstrates that integrating diabetes screening into ophthalmologic care is not only clinically justified but also economically efficient, especially in resource-limited contexts like Mexico.

The findings also emphasize the importance of cross-specialty collaboration between general practitioners and ophthalmologists to reduce or eliminate sight-threatening DR among type 2 diabetes patients. A preventive approach, based on early diagnosis, strict glycemic control, and regular eye examinations, should be prioritized over reactive or late-stage care. Moreover, public health strategies should strengthen community awareness, equitable access to screening, and continuity of care, ensuring that patients identified through programs like APEC’s are effectively linked to primary and secondary prevention services. Given that diabetes remains the second leading cause of death in Mexico and the primary cause of disability, expanding early detection programs could generate substantial population-level benefits. Integrating ophthalmologic screening with systemic health interventions creates a unique opportunity to bridge diagnostic gaps, foster patient empowerment, and reduce both the clinical and economic burden of diabetic complications.

Despite these promising results, the study has several limitations. First, the baseline assumption that 74.2% of diagnosed individuals will seek treatment is based on observed cross-sectional patterns and may not fully capture behavioral responses over time. Second, our economic valuation is anchored on a single country-level estimate of the Value of a Statistical Life (VSL) for Mexico, which could vary based on methodological updates, macroeconomic conditions, or subnational characteristics. Lastly, the study does not include a long-term follow-up of clinical outcomes, limiting our ability to directly measure changes in health trajectories. Future research could address these limitations by tracking patient outcomes over time, identifying barriers to treatment adherence, and refining estimates of treatment uptake across different demographic and socioeconomic groups.

In summary, APEC’s early screening model exemplifies how targeted interventions within specialized institutions can yield substantial health and economic benefits. Its dual role in preserving vision and enabling chronic disease detection can improve individual well-being while reducing pressure on the healthcare system. These results support the case for scaling similar initiatives and strengthening public–private efforts to address Mexico’s growing diabetes epidemic. Ultimately, an integrated, multidisciplinary, and preventive approach, where ophthalmologists, primary care physicians, and public health institutions collaborate, is essential to curb the rising tide of diabetes-related blindness and disability in Mexico. Strengthening early screening initiatives like APEC’s represents a strategic, high-impact investment in both population health and social welfare.

## Figures and Tables

**Table 1:** Descriptive Statistics of First-Time Patients Screened at APEC Hospital, 2023.

Variable	Observations	Mean	Std. Dev	95% Confidence Interval
Woman ^1^	25,411	0.63	0.48	0.63–0.64
Age (years)	25,411	55.09	18.74	54.86–55.32
Height (meters)	12,512	1.59	0.14	1.589–1.594
Weight (kilograms)	12,144	70.56	15.79	70.28–70.84
Body Mass Index (kgs/m^2^)	9501	28.20	16.07	27.88–28.52
Heart Rate (beats per minute)	21,121	75.08	12.48	74.91–75.25
Respiratory Rate (breaths per minute)	13,240	22.35	17.88	22.05–22.66
Capillary glycemia (mg/dL) ^2^	15,081	129.98	72.34	129.46–131.76
Capillary glycemia > 200 ^3^	15,081	0.12	0.32	0.11–0.12

Source: Authors’ own elaboration based on APEC administrative data. Notes: ^1^ Dummy variable equal to 1 if female and 0 if male. ^2^ Taken at any time of day regardless of the interval since the last food intake. ^3^ Dummy variable equal to 1 if the individual has a glucose record greater than 200 mg/dL.

**Table 3:** Sensitivity Analysis.

Case		Parameter	*BCR_APEC_*			*BCR_FULL_*		
			Mean	Mean Difference vs. Baseline Scenario (%)	95% Confidence Interval	Mean	Mean Difference vs. Baseline Scenario (%)	95% Confidence Interval
(a)	Probability of initiating treatment after diagnosis or notification of uncontrolled diabetes	10%	73.1	−86.5	69.76–76.41	8.9	−0.8	8.85–8.93
		25%	182.7	−66.3	174.40–191.04	8.9	−0.2	8.89–8.98
		50%	365.4	−32.6	348.81–382.07	9.0	−0.1	8.91–9.00
		100%	730.9	34.8	697.61–764.14	9.0	0.0	8.92–9.01
(b)	Disability weight	0.17	377.6	−30.4	360.45–394.83	6.2	−30.4	6.21–6.27
		0.32	708.0	30.6	675.80–740.25	11.7	30.6	11.64–11.75
(c)	Discount rate	3%	980.2	80.8	934.54–1025.93	9.0	0.0	8.92–9.01
		5%	805.8	48.6	768.63–842.93	9.0	0.0	8.92–9.01
		15%	400.8	−26.1	382.66–418.97	9.0	0.0	8.91–9.00
(d)	Value per Statistical Life (VSL) for Mexico (million USD)	$1.0	271.2	−50.0	258.81–283.50	4.5	−50.0	4.46–4.50
		$1.5	406.7	−25.0	388.22–425.24	6.7	−25.0	6.69–6.75
		$2.5	677.9	25.0	647.04–708.74	11.2	25.0	11.14–11.25
Average			498.5	−8.1		8.6	−98.4	
Std. Dev.			276.1			2.0		
Minimum			73.4	−86.5		4.5	−50.0	
Maximum			980.2	80.8		11.7	30.6	

## Data Availability

The data are not publicly available due to ethical, legal, or other concerns.
